# Simulations and Experimental Analysis of a High Viscosity Inkjet Printing Device Based on Fabry–Pérot Resonator

**DOI:** 10.3390/s22093363

**Published:** 2022-04-27

**Authors:** Muhammad Ali Shah, Duck-Gyu Lee, Youngsoo Kim, Shin Hur

**Affiliations:** 1 Korea Institute of Machinery and Materials, Daejeon 34103, Korea; ali@kimm.re.kr (M.A.S.); educk9@kimm.re.kr (D.-G.L.); ys0076@kimm.re.kr (Y.K.); 2 Department of Nano-Mechatronics, University of Science and Technology, Daejeon 34113, Korea

**Keywords:** Fabry–Pérot resonator, standing waves, acoustic pressure, high viscosity

## Abstract

The study investigates the effect of changing various input parameters on the pressure responses at acoustic cavities of a droplet-based acoustic printing device consisting of a Fabry–Pérot (FP) resonator and a standing wave-source chamber. The standing wave of the acoustic radiation pressure at the FP resonator is analyzed. The behavior of the standing wave and acoustic radiation force at the FP resonator is presented and compared with the measured results by varying the position of the standing wave-generating plate. The pressure changes inside the standing wave-source chamber are investigated and discussed to determine the reason for the sudden high-pressure drop at the FP resonator. Furthermore, the effects of inserting the nozzle and droplet inside the FP resonator on the standing wave and acoustic radiation force are analyzed. Experimental analysis is performed by collecting acoustic pressure data at the outlet of the FP resonator. The simulated and measured pressure drop behaviors are compared. The presented numerical approach can be used to set optimal design guidelines for obtaining a higher acoustic pressure inside the acoustic cavities of droplet-based acoustic jetting and other acoustofluidic devices.

## 1. Introduction

Droplet-based inkjet printing is a well-established technology that has been applied in diverse areas, such as textiles [1], light-emitting displays [2], flexible electronics [3], micro lenses [4], pharmaceuticals [5], and cell structures [6]. It is characterized by extreme reproducibility, high drop productivity, and a small volume. Various inkjet printing technologies have been developed [7,8,9,10,11,12], among which piezoelectric inkjet printing is the most reliable and developed technology [12]. However, the piezo-driven inkjet printing technique fails to print inks with extremely high viscosity; therefore, transfer techniques such as electrohydrodynamic jet printing [13], laser-based printing [14], needle dispensers [15], and aerosol-jet printing, are essential in addressing this limitation [16]. However, various parameters must be optimized in these high viscosity ink printing techniques for various ink compositions, which makes it difficult for materials whose physical properties change over time. Another approach is to use higher acoustic radiation forces to jet high viscosity ink. Acoustic cavities with standing acoustic waves can be employed to further enhance the acoustic force. One major advantage of acoustic forces is that they have no direct contact with the ink droplets.

Acoustic radiation forces and standing acoustic waves are mostly used in various acoustofluidic fields [10,17,18,19,20], especially in surface acoustic wave (SAW) printing [21,22] and acoustic levitation [23,24]. In SAW printing, high-frequency SAW on the surface of the liquid generates an acoustic radiation force that propels the droplet. Acoustic levitation refers to the suspension of objects in midair by acoustic radiation. In acoustic levitation, a standing wave is established between an emitter and a reflector, where acoustic pressure nodes are used to suspend the matter in the levitator. This concept has been widely used in various applications involving various design concepts. The transport and mixing of multi-droplets via ultrasonic arrays have been demonstrated in previous studies [25,26]. Naka and Hasegawa demonstrated the breakup of a levitated droplet in a single acoustic levitator [27]. Acoustic levitation has been used in self-assembly [28]. In the acoustic levitation technique, the suspension of droplets is analyzed in the acoustic nodes of standing waves. The antinode of the standing wave can be used to jet an ink droplet pendant at the nozzle exit.

Researchers from Harvard University recently developed an acoustophoretic printing technique for printing high viscosity inks [29]. The nozzle outlet was placed in the Fabry–Pérot (FP) resonator at a location with a higher-pressure response (at the antinode of the standing wave). In their study, the actuator was driven at a frequency of 25 kHz to generate a standing wave in an FP resonator. The standing wave generated a higher force compared to other printing techniques. This force is known as the acoustic radiation force exerted on the pendant droplet at the nozzle outlet, which causes the droplet to jet. They printed various high viscosity materials using this technique. They analyzed parameters such as nozzle diameter, acoustic radiation force, and distance from the substrate to the nozzle outlet. However, there is room for improvement and, recently, investigation of this has developed printing techniques.

In this study, we developed a printing system similar to that proposed in [29]. We named the printing device the standing wave amplification module (SWAM). SWAM was developed at a frequency of 18 kHz. SWAM comprises a standing wave-source chamber, FP resonator, nozzle, standing wave generating plate (SWGP), and actuator. The actuator is placed at one end and the FP resonator is drilled at the other end. Extensive numerical simulations and experimental analyses were conducted. The acoustic pressure responses in the standing wave source chamber and FP resonator were extracted using numerical simulations. The SWGP was actuated at different positions, and its effects on the output pressure were analyzed. Additionally, the effects of varying the droplet size and nozzle shape on the pressure response were analyzed. An experimental analysis was performed by collecting the data of the acoustic pressure response at the outlet of the FP resonator, and the changing behavior of the simulation results were compared with the measured results. To the best of our knowledge, this analysis is presented for the first time. It will provide insight into the FP resonator-based acoustophoretic printing technique for the research community working on this specific high viscosity inkjet printing technology. Sensors are used in various applications and play a vital role in our daily life. Metals are used for the transduction mechanism in sensors. The high viscosity inks of metals can be printed using acoustic-based the high viscosity acoustophoretic inkjet printing mechanism. Furthermore, packaging and bio-printing technologies are also related to high viscosity inks. To print high viscous inks, a much higher force is needed to propel the droplet at the nozzle exit. The proposed approach can be used to set optimal design guidelines to obtain a higher-acoustic pressure, which can lead to a higher jetting force inside acoustic cavities of acoustophoretic printing technology.

The remainder of this paper is organized as follows. Section 2 presents the theory of acoustic radiation pressure and force and the method of numerical simulations. Section 3 presents extensive numerical simulations of the standing wave-source chamber and FP resonator. Section 4 presents the experimental analysis. Section 5 concludes the paper.

## 2. Theory and Method

**Figure 1:**
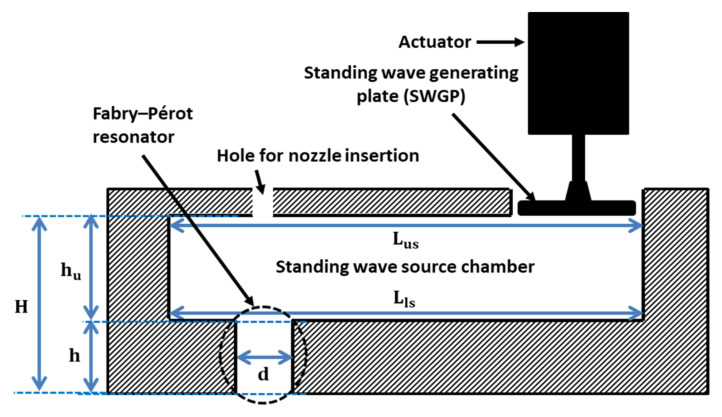
Schematic cross-sectional representation of SWAM, in which, ${\;L}_{\mathrm{us}}$ and $L_{\mathrm{ls}}$ represents the upper and lower surface lengths of the standing wave-source chamber, respectively, ${\;h}_{u}$ and h represents the heights of standing wave-source chamber and FP resonator, respectively, and H represents the height of both the standing wave-source chamber and FP resonator.

**Figure 2:**
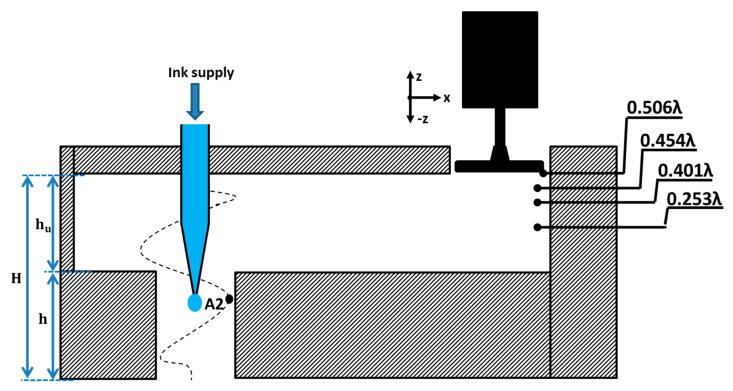
Schematic cross–sectional representation of SWAM with nozzle and standing wave at acoustic cavity of height H and standing wave-source chamber showing four different positions at which the SWGP is actuated.

**Figure 3:**
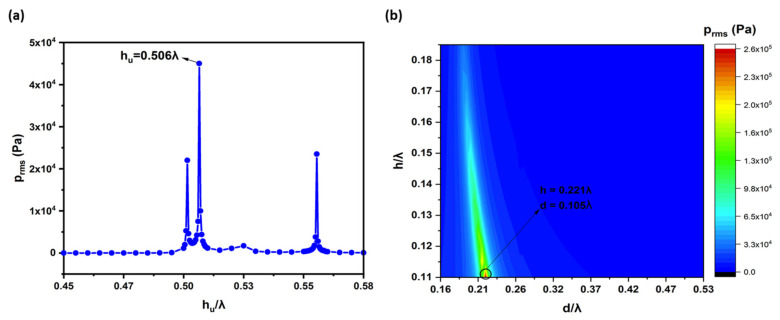
Optimized acoustic cavities for higher RMS pressure, P_rms_ of (**a**) upper acoustic chamber height,${\;h}_{u}$, and (**b**) FP resonator height, h and diameter, d.

A cross-sectional schematic of SWAM is shown in Figure 1. A standing wave-source chamber was used to create a standing wave using an actuator. The SWGP was attached to the actuator. When the actuator was activated, the SWGP vibrated, generating a standing wave in the standing wave-source chamber and amplifying a standing wave inside the FP resonator. An FP resonator is an amplifying medium used to enhance the pressure response. We optimized the FP resonator for a driving frequency of 18 kHz. Figure 2 shows the cross-sectional representation of the SWAM device in which the nozzle was inserted in the FP resonator. The nozzle outlet was placed at a location where there was maximum pressure (at antinode A2 of the standing wave). First, the standing wave-source chamber height (h_u_) was optimized by reporting the peak of the root mean square (RMS) pressure (P_rms_) within the standing wave-source chamber. The reported maximum P_rms_ versus h_u_ graph is shown in Figure 3a, where h_u_ = 0.506λ = 9.63 mm provides the maximum pressure response. The optimized diameter (d) and height (h) of the FP resonator were achieved using an approach reported in references [29,30]. Figure 3b shows the contour plots from h–d. We obtained h = 0.221λ = 4.2 mm and d = 0.105λ = 2 mm. The geometric, acoustic, and model parameters are listed in Table 1.

The acoustic standing wave was generated at the cavity with height H owing to the traveling and reflection of acoustic waves in the standing wave-source chamber. The standing wave generates the acoustic radiation force on the pendant droplet at the nozzle exit. Figure 2 shows that placing the nozzle outlet at antinode A2 of the standing wave forced the droplet to jet. The radiation pressure $P_{rad}$ exerted on the droplet is given by the following equation:

(1) $$P_{\mathrm{rad}}=\left(\frac{p^{2}{}_{\mathrm{rms}}}{2\rho_{0}c_{0}{}^{2}}-\rho_{0}\frac{v^{2}{}_{\mathrm{rms}}}{2}\right)$$

where $p_{\mathrm{rms}}$ and $v_{\mathrm{rms}}$ are the RMS pressure and particle velocity of the host-acoustic medium (air), and $\rho_{0}$ and $c_{0}$ are the density and speed of sound of the host medium, respectively. By integrating the acoustic radiation pressure over the entire surface S of the droplet, the acoustic radiation force can be calculated [29].

(2) $$F_{\mathrm{rad}}=\int_{S}^{.}P_{\mathrm{rad}}\overset{\to }{n}\mathrm{dS}$$

In this study, the theory of acoustic radiation pressure and force was applied to the presented model, and some of the numerically simulated results were compared with the measured results. The commercial software COMSOL Multiphysics (4.3b, COMSOL Inc., Stockholm, Sweden) was used to perform numerical simulations for predicting the RMS pressure, RMS velocity, acoustic radiation pressure, and acoustic radiation force. The simulations were performed in the three-dimensional (3-D) domain. Three studies were conducted: varying the dimensions and position of the nozzle, position of the SWGP, and droplet shape. Figure 2 shows the SWGP positions where the pressure responses at the standing wave-source chamber and FP resonator were analyzed by adjusting the SWGP at specified positions of 0.506λ, 0.454λ, 0.401λ, and 0.253λ.

## 3. Numerical Simulations

**Figure 4:**
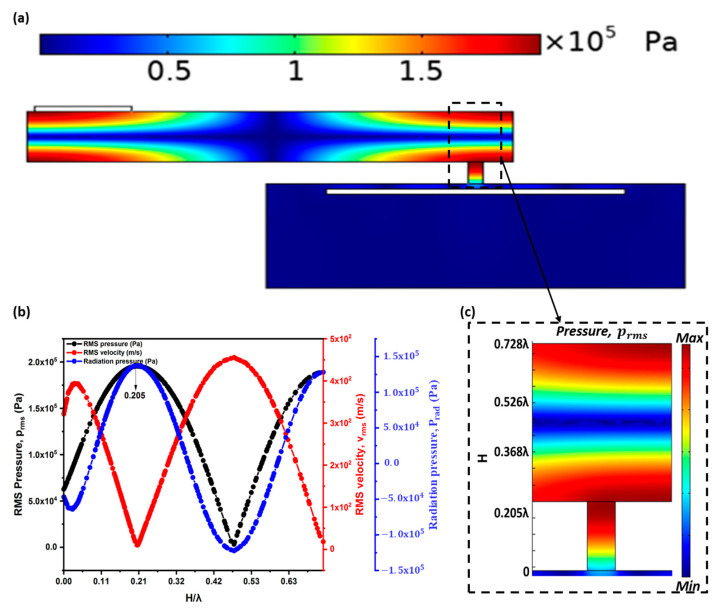
Numerical simulation results at acoustic cavity of height, H of (**a**) RMS pressure, velocity, and radiation-pressure responses, (**b**) RMS pressure distribution, (**c**) RMS pressure at acoustic cavity of height, H where the nozzle outlet has to be placed at H = 0.205λ within the Fabry–Pérot resonator.

The physics of the acoustic–structure interaction was used to perform numerical simulations in the commercial software COMSOL Multiphysics. A COMSOL, ‘Pressure Acoustics, Frequency Domain’ interface was coupled in a Multiphysics domain with the ‘Solid Mechanics’ interface. This acoustic–structure interaction couples the acoustic medium with a solid. A 3-D model was used to analyze the acoustic field inside the FP resonator and standing wave-source chamber. A frequency of 18 kHz was used for all the simulations, and the acoustic chamber dimensions were scaled with the wavelength. From the optimized acoustic chambers, the RMS pressure ($p_{\mathrm{rms}}$), acoustic radiation pressure ($P_{\mathrm{rad}}$), and RMS velocity ($v_{\mathrm{rms}}$), were acquired at an acoustic cavity of height H. The predicted standing wave pressure and particle velocity profiles are shown in Figure 4a. The pressure and particle velocity are in antiphase to each other. A typical pattern of the RMS pressure distribution is shown in Figure 4b. The pendant droplet can be ejected by a high acoustic radiation force by positioning the nozzle exit at 0.205λ (where there is maximum pressure within the FP resonator). Droplet jetting was already reported in reference [29]. This study investigates the pressure responses and acoustic radiation force within the acoustic cavities, which provides a platform for the research community working in the acoustofluidics and acoustic–structure interaction fields. Particularly, droplet manipulation inside the acoustic cavities with standing waves could benefit from this study.

### 3.1. Effect of Nozzle and Droplet

**Figure 5:**
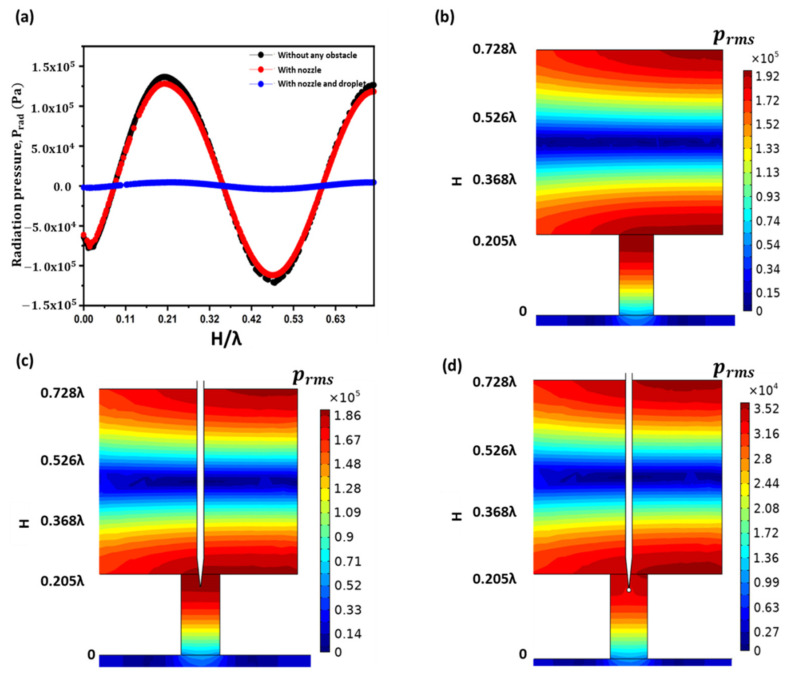
Numerical simulations of (**a**) standing wave of radiation pressure at acoustic cavity of height, H without any obstacle, with only nozzle, and with the nozzle and droplet, and RMS pressure distribution at acoustic cavity of height, H (**b**) without any obstacle, (**c**) with nozzle only, and (**d**) with the nozzle and droplet.

**Figure 6:**
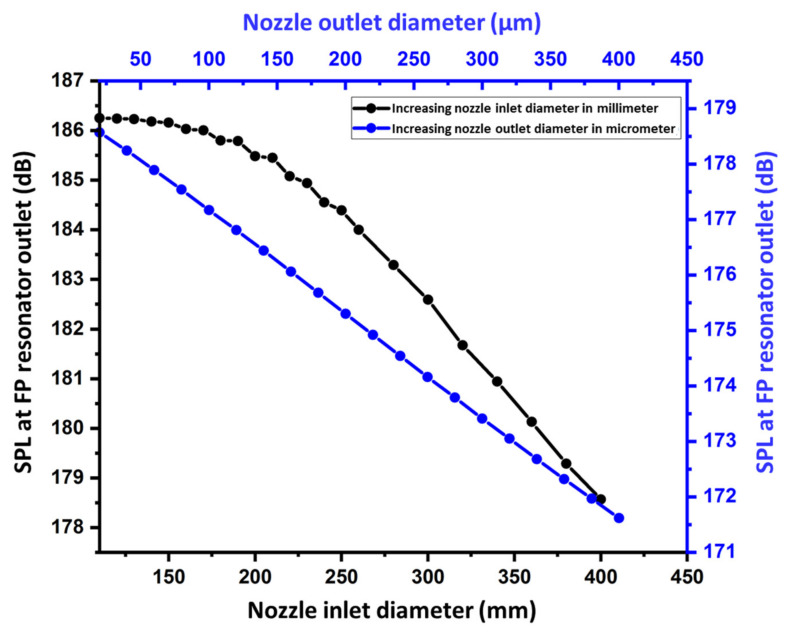
Effect of changing the nozzle inlet and outlet diameters on the pressure response at the FP resonator outlet.

The pressure responses within the acoustic cavity of height H were numerically calculated with and without an obstacle (nozzle and droplet). Figure 5 shows the numerically predicted effect of an obstacle on acoustic radiation pressure. The presence of an obstacle affected the amplitude of the antinodes. It did not affect the nodes or the position of the standing wave. The amplitude of the acoustic wave antinode decreased by inserting only the nozzle inside the FP resonator. There was a larger reduction in the antinode amplitude when a spherical droplet of water was attached to the nozzle exit. Therefore, the insertion of the droplet must be considered for numerical simulation analysis to obtain optimized parameters. Thus, the appropriate input-optimized parameters can be achieved for droplet jetting. Figure 5a shows a comparative analysis of the standing wave radiation pressure without any obstacle, with only the nozzle, and with the nozzle and sphere droplet. The RMS pressure distribution within the acoustic cavity of height H without any obstacle, with only the nozzle, and with the droplet and nozzle, is shown in Figure 5b–d. From Figure 5c,d, the maximum RMS pressure is significantly disturbed when the droplet and nozzle are placed inside the FP resonator compared to when only the nozzle is placed. The effect of changing the nozzle inlet and outlet diameters on the sound pressure level (SPL) at the FP resonator outlet is also investigated and is shown in Figure 6. In both the cases, by increasing the nozzle diameter, the pressure decreases. This decrease in pressure response is due to the increasing of resistance to the air flow in-between the nozzle and FP resonator walls.

### 3.2. Effect of Changing SWGP Position

**Figure 7:**
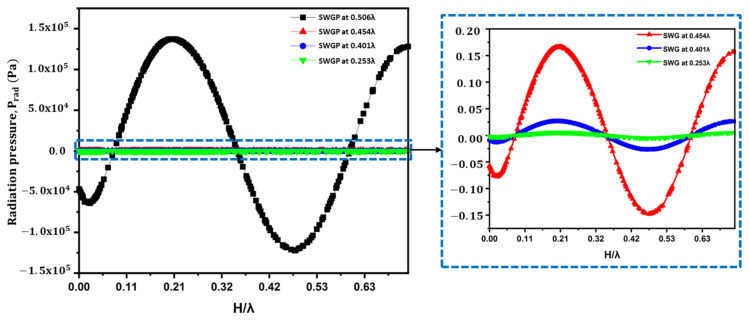
Numerical simulation results of standing wave radiation pressure at acoustic cavity of height, H when the SWGP was actuated at 0.506λ (upper surface of the standing wave-source chamber), 0.454λ, 0.401λ, and 0.253λ.

**Figure 8:**
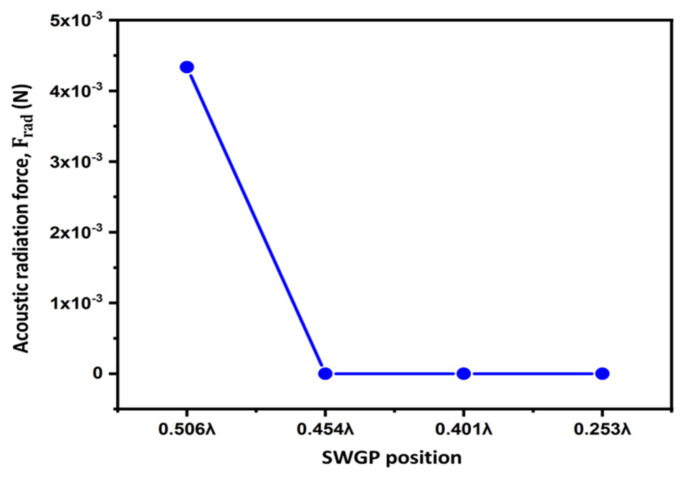
Acoustic radiation force on sphere droplet inside the FP resonator when the SWGP was actuated at 0.506λ, 0.454λ, 0.401λ, and 0.253λ.

**Figure 9:**
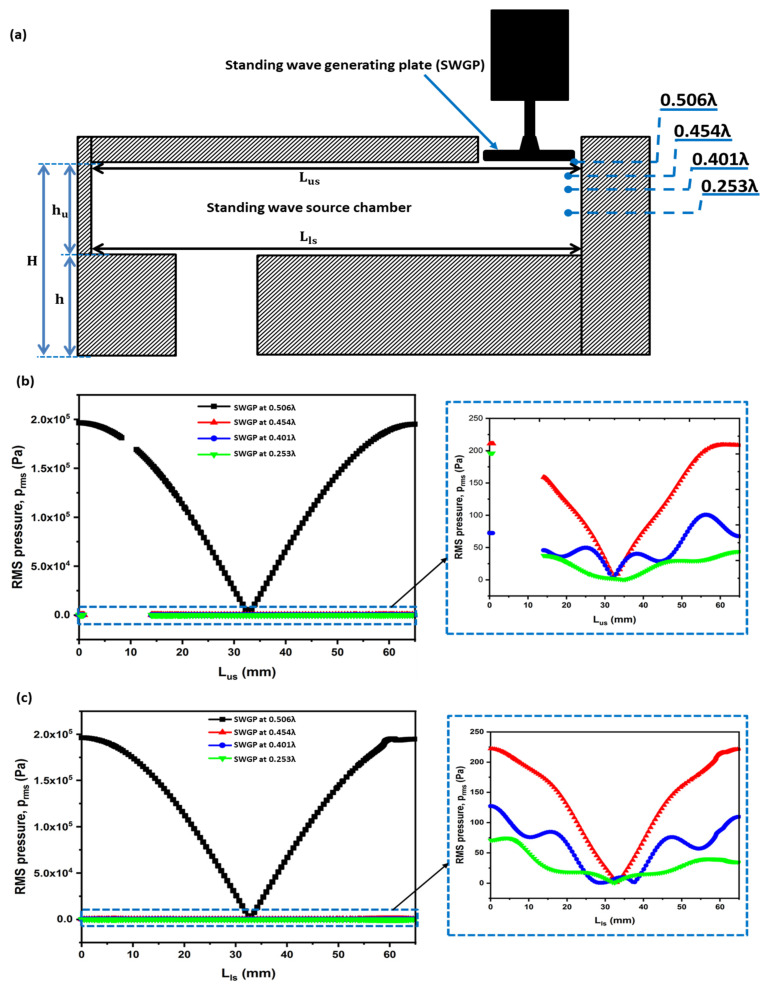
(**a**) Schematic cross-sectional representation of SWAM showing four different positions at which the SWGP is actuated and numerical simulation results of RMS pressure across the (**b**) upper surface length (L_us_) and (**c**) lower surface length (L_ls_) of the standing wave-source chamber when the SWGP was actuated at 0.506λ, 0.454λ, 0.401λ, and 0.253λ.

**Figure 10:**
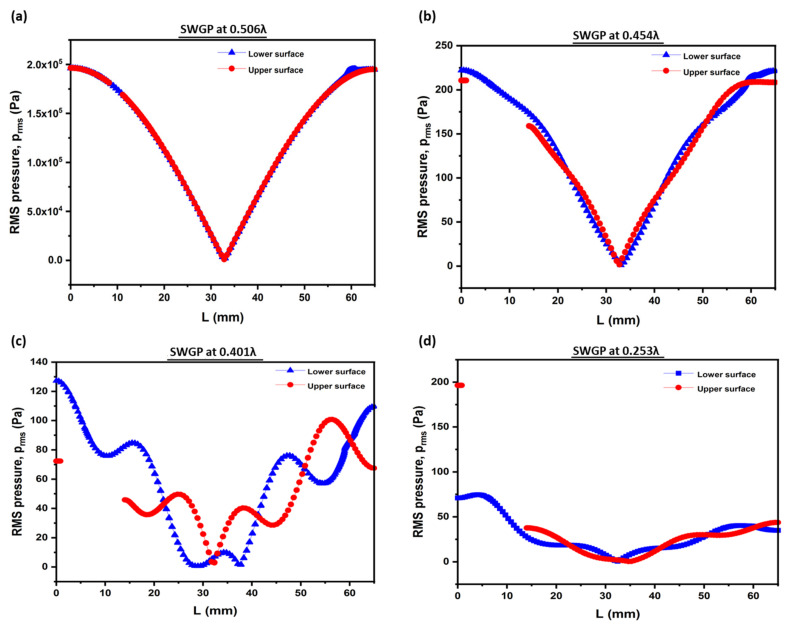
Comparison of the RMS pressure of the upper and lower surfaces of standing wave-source chamber when (**a**) SWGP was actuated at 0.506λ, (**b**) SWGP was actuated at 0.454λ, (**c**) SWGP was actuated at 0.401λ, and (**d**) SWGP was actuated at 0.253λ.

Optimizing the position of the SWGP is expedient. The standing acoustic waves are affected by changes in the SWGP position. We analyzed the standing acoustic waves within the FP resonator and in the standing wave-source chamber by changing the position of the SWGP. Figure 2 shows a schematic representation of the SWGP positions in the standing wave-source chamber. The standing waves were analyzed by adjusting the SWGP to positions 0.506λ (SWGP at the upper surface of the standing wave-source chamber), 0.454λ (SWGP moved 1 mm downward from the upper surface), 0.401λ (SWGP moved 2 mm downward from the upper surface), and 0.253λ (SWGP moved to the center). We analyzed the standing wave in the acoustic cavity of height H and the RMS pressure in the standing wave-source chamber. Figure 7 shows the standing wave of the radiation pressure. A considerable reduction in the acoustic radiation pressure occurred when the SWGP was moved to 0.454λ. The pressure decreased by further moving it down to 0.401λ and 0.253λ. Figure 8 shows a similar reduction trend for the acoustic radiation force acting on the spherical droplet when the SWGP was actuated at the four different positions in the standing wave-source chamber. The pressure reduction trend is experimentally verified in Section 4. The RMS pressure in the standing wave-source chamber was investigated across the lengths of the upper and lower surfaces to understand the sudden reduction in the acoustic radiation pressure and force. The lengths of the upper and lower surfaces were denoted as L_us_ and L_ls_, respectively, as shown in Figure 9a. The RMS pressure responses across L_us_ and L_ls_ are shown in Figure 9b,c, respectively. The blank area between 2 mm and 14 mm in Figure 9b is due to the actuating of SWGP placed at that specific location across L_us_. A considerable pressure reduction occurred when the SWGP position was moved in the downward (*z*-axis) direction. Furthermore, nodes and antinodes were observed when the SWGP was placed at the 0.454λ, 0.401λ, and 0.253λ positions. Figure 10 shows a comparison of the pressures at the actuating positions. The RMS pressure response was similar in both the upper and lower surfaces of the standing wave-source chamber at position 0.506λ (SWGP at the upper surface of the standing wave-source chamber), as shown in Figure 10a. However, when the SWGP was actuated at positions 0.454λ, 0.401λ, and 0.253λ, further nodes and antinodes were observed, and the antinodes of the RMS pressure at both surfaces became antiphase to each other, as shown in Figure 10b–d respectively. This antiphase behavior of the RMS pressure in the standing wave-source chamber caused a sudden reduction in the standing wave radiation pressure at the FP resonator.

## 4. Experimental Analysis

**Figure 11:**
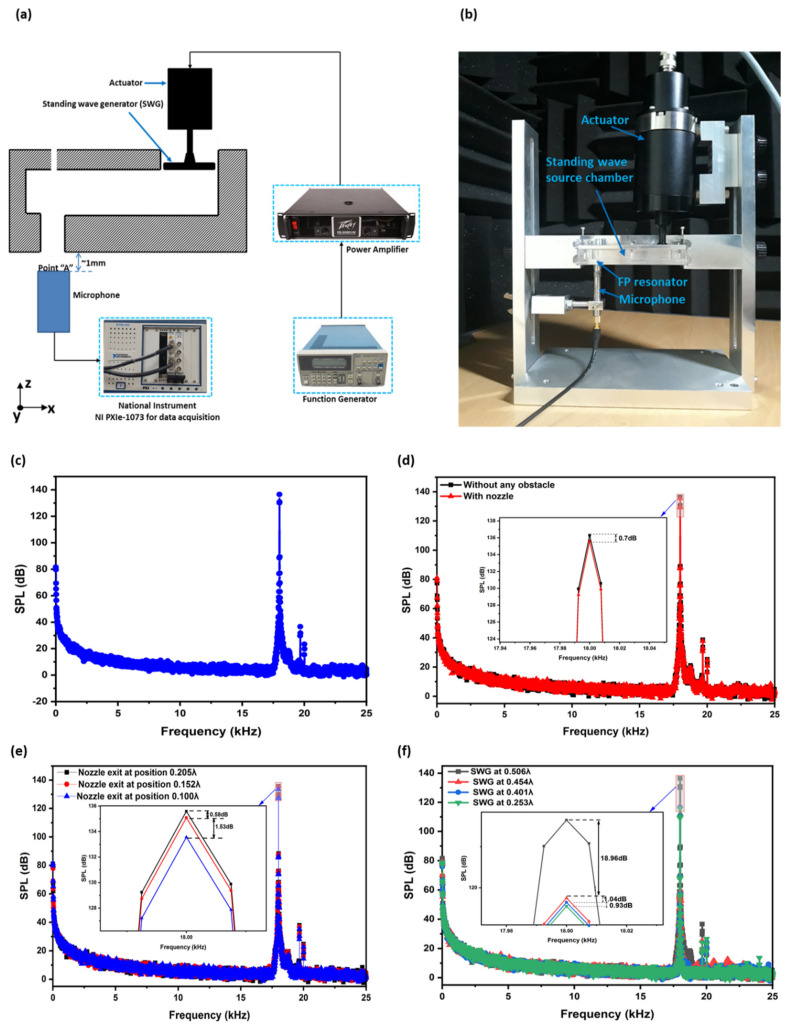
Experimental setup for SPL measurement. (**a**) Schematic representation, (**b**) setup of fabricated FP resonator-based printing device, actuator, and microphone for measuring SPL, (**c**) measured results of SPL at point A, (**d**) comparison of SPL at point A without any obstacle and with conical nozzle, (**e**) comparison of SPL at point A when the nozzle exit is placed at three different positions inside the FP resonator, and (**f**) comparison of SPL at point A when the SWGP was actuated at four different positions.

In this section, the experimental setup developed to measure the sound pressure level (SPL) is described, and the results are discussed. A sinusoidal wave with a frequency of 18 kHz was generated using a function generator and amplified by a power amplifier (Peavey CS 8080 Hz). The amplifier was operated at an RMS voltage swing of 115 volts. An ultrasonic actuator (CU18A) was used to drive SWGP. A microphone was placed 1 mm from the surface of the FP resonator outlet to measure the acoustic pressure. This location was named point A. A national instrument device (NI PXIe-1073) was used for the data acquisition. The overall experimental schematic representation is shown in Figure 11a, and the implemented setup and fabricated device are shown in Figure 11b.

We measured the SPL at point A to verify the numerical simulation results of the FP resonator and standing wave-source chamber at 18 kHz. The actuator was actuated at a resonant frequency of 18 kHz. The sine-wave signal was amplified using a power amplifier. First, the SPL was measured at point A without a nozzle. Figure 11c shows a higher SPL response at 18 kHz. We inserted a conical nozzle (without any ink) inside the FP resonator at a location where there was a higher pressure response to observe the effect of the nozzle. SPL was measured at Point A. A comparative analysis of the SPL was performed in Figure 11d with and without a nozzle. The reduction of 0.7 dB SPL was observed by inserting a nozzle at the maximum pressure location inside the FP resonator. A reduction of 0.58 and 1.53 dB SPL was observed by further moving the nozzle in downward direction (z direction), that is, 0.152λ and 0.100λ positions, as shown in Figure 11e.

The simulation results regarding the effect of changing the SWAM position on the pressure response were verified by an experimental approach. The SWGP was actuated at 0.506λ, 0.454λ, 0.401λ, and 0.253λ, and the SPL was measured at point A. Figure 11f shows the measured SPL at point A when the SWGP was actuated at the four positions. A considerable reduction of 18.96 dB SPL, which is an extremely high pressure in Pascal, was observed when the SWGP was placed and actuated at 0.454λ. By further moving the SWGP down and actuating it at 0.401λ and 0.253λ, the SPL reduced (1.04 and 0.93 dB respectively); however, it was less than when the SWGP was moved to 0.454λ. This SPL reduction trend validates the numerical simulation results.

## 5. Conclusions

In this study, a recently developed FP resonator-based acoustophoretic printing device for printing high viscosity inks was further investigated for new findings in terms of acoustic radiation pressure and force. Through extensive numerical simulations, the acoustic pressure responses and acoustic radiation force inside the acoustic cavities were extracted. The effects of changing the SWGP position and droplet and nozzle shapes on the acoustic radiation force and amplitude of the antinode of the standing wave were investigated. Numerical simulations and measurement results revealed that when pressurizing the standing wave-source chamber at a location lower than the upper surface of the standing wave-source chamber, there is a higher pressure drop at the FP resonator. By investigating the acoustic pressures at the upper and lower surfaces of the standing wave-source chamber, it was concluded that the nodes and antinodes of the pressure waves increased, and the antinodes at both surfaces became antiphase to each other when the SWGP was actuated at a location lower than the upper surface of the standing wave-source chamber. This antiphase behavior may cause a sudden higher pressure reduction in the standing wave at the FP resonator. The standing wave was investigated by inserting a nozzle and droplet inside the FP resonator. It was concluded that the pressure could be reduced by inserting an obstacle inside the FP resonator. The pressure was further reduced by moving the conical nozzle in the downward direction (z direction). There was an extremely high pressure reduction when a droplet was attached to the exit of the conical nozzle. Therefore, for the optimization of FP resonator-based acoustophoretic printing device or other droplet-based acoustofluidic devices, it is recommended that the droplet be numerically analyzed to obtain the optimized parameters.

## Figures and Tables

**Table 1:** Acoustical and model parameters of the SWAM.

Parameters	Values
Frequency (f)	18 kHz
Wavelength (λ)	19 mm
Standing wave source chamber length (L)	65 mm
$\mathrm{Standing}\;\mathrm{wave}\;\mathrm{source}\;\mathrm{chamber}\;\mathrm{height}\;(h_{u})$	0.506λ
FP resonator height (h)	0.221λ
FP resonator diameter (d)	0.105λ
$\mathrm{Air}\;\mathrm{density}\;(\rho_{0}$)	$1.18\;\mathrm{kg}/m^{3}$
$\mathrm{Speed}\;\mathrm{of}\;\mathrm{sound}\;(c_{0}$)	343 m/s

## Data Availability

Not applicable.
